# Borderline Personality Disorder “Discouraged Type”: A Case Report

**DOI:** 10.3390/medicina58020162

**Published:** 2022-01-21

**Authors:** Lavinia Duică, Elisabeta Antonescu, Maria Totan, Gabriela Boța, Sînziana Călina Silișteanu

**Affiliations:** 1 Faculty of Medicine, “Lucian Blaga” University of Sibiu, 550169 Sibiu, Romania; lavinia.duica@ulbsibiu.ro (L.D.); maria.totan@ulbsibiu.ro (M.T.); gabriela.bota@ulbsibiu.ro (G.B.); 2 “Dr. Gheorghe Preda” Clinical Psychiatric Hospital, 550082 Sibiu, Romania; 3 Faculty of Medicine and Biological Sciences, “Stefan cel Mare” University of Suceava, 720229 Suceava, Romania; sinziana.silisteanu@usm.ro

**Keywords:** Borderline Personality Disorder, depression, perfectionism, obsessive-compulsive symptoms, fearfulness, dependency

## Abstract

Borderline Personality Disorder (BPD) is a mental illness associated with a significant degree of distress and impairment because of the difficulties in effectively regulating emotions. BPD is frequently associated with Depressive Disorders, most commonly Major Depressive Disorder and Dysthymia. Here, we present a case report of an 18-year-old female patient hospitalized with a severe depressive episode and psychotic symptoms. A few months after discharge, the interpersonal difficulties, unstable self-image, fear of chronic abandonment, feeling of emptiness, paranoid ideation, helplessness, obsessive-compulsive elements, perfectionism, and social retreat led to the patient’s impaired functionality. The spectrum of signs and symptoms presented were characteristic of BPD. The specific presentation of mixed dependent/avoidant pattern of personality, with persistent feelings of guilt and shame, social anxiety, emotional attachments, obsessions, and feelings of inadequacy have further narrowed the diagnosis to discouraged BPD, as described by Theodore Millon. In our case, this particular subtype of personality disorder can be understood as BPN associated with social perfectionism. Both BPD and perfectionism, as a trait personality, were thought to exacerbate issues with self-conception and identity formation in this patient.

## 1. Introduction

Borderline Personality Disorder (BPD) is associated with significant emotional suffering and functional impairment [1], including low occupational and educational attainment, difficulty in forming long-term relationships, increased partner conflict, sexual risk-taking, low levels of social support, low life satisfaction, and increased service use [2]. The most frequently co-occurring psychiatric disorders with BPD are Depressive Disorders (DD), most commonly Major Depressive Disorder (MDD) and Dysthymia. Between 70 and 90% of individuals with BPD go through at least one major depressive episode or exhibit another depressive disorder throughout the course of their lives [3]. According to Linehan’s biopsychosocial model, BPD has also been associated with childhood maltreatment and an invalidating family environment [4]. Indeed, childhood maltreatment, including family adversity, exposure to physical and sexual abuse, or neglect, has been found to be a robust and predictive risk factor for BPD [5].

The high prevalence of comorbid pathology amongst patients with BPD is widely recognized [6]. Therefore, a large variation in the expression of BPD pathology is apparent in clinical practice. For example, Critchfield et al. [7] describe three subtypes of BPD patients: those with co-occurring cluster A Personality Disorder (PD) traits (elevated schizotypal and paranoid features), those with cluster B traits of PD (elevated narcissistic and histrionic features), and those with cluster C traits of PD (elevated avoidant and obsessive-compulsive features). In Theodore Millon’s evolutionary model of personology, subtypes describe variations of each of the prototypical personality disorders derived from research and clinical observation [8]. Each subtype, or prototypical variant, shares the core features of the main prototype with one, two, or three other different PD. Millon’s four subtypes of Borderline Personality Disorder are: discouraged, self-disruptive, impulsive, and petulant.

In a recent study, the profile of the “inhibited” subtype resembled the “Discouraged” subtype characterized by avoidant, dependent features and unexpressed anger. These patients internalize more, are less impulsive, and are less likely to communicate their emotions [9]. The case brought to our attention was a patient who presented to the hospital with a recurrent, major depressive episode with psychotic symptoms, who, after the resolution of the acute symptomatology, was diagnosed with BPD, discouraged type. This is an interesting clinical case that resembles both features of cluster B Personality Disorder (dramatic, emotional, or erratic disorders) and perfectionism, a trait associated with cluster C Personality Disorder (anxious or fearful disorders) [1] as seen from the psychopathological features presented below. This combination posed significant challenges in accurately determining diagnosis and will aid clinicians in treatment recommendation.

## 2. Case Presentation

An 18-year-old female patient, from the rural region of Romania, was brought by her father to the emergency department for severe anxiety, suspiciousness, weight loss, food negativism, complex auditory and visual hallucinations, and fragmentary paranoid ideas. At age 16, the patient had a hospital admission to the pediatric psychiatric department with depressive and obsessive-compulsive symptoms and general low functionality in daily and school activities. She was diagnosed with a severe depressive episode and obsessive-compulsive disorder (OCD). At age 17, she was again diagnosed as an outpatient with OCD for obsessive-compulsive symptoms, low functionality, and school dropout. She was treated with Olanzapine 15 mg; however, after a few weeks, the treatment was discontinued at the request of her father because he considered that the treatment was “too strong”, and that his daughter did not need a psychiatric treatment, although there were no adverse reactions to the drug.

Life and family history showed that the patient lived in the rural area with her parents and two brothers with whom she had relatively harmonious relationships. She studied for three years at the High School for Arts, painting section, and, at some point, she developed a good reputation for her artistic talent. Her father told us that he considered that a major trauma in his daughter’s life was her deep disappointment at the inability of her family to afford the financial costs for studying anime in Japan, which was her exclusive life project during that period of time. When admitted to the hospital, the patient had no occupation and had dropped out of school for the past year.

Sometime after her first admission to the adult psychiatric ward, the patient communicated to us some information about her life history. She had tense interactions with her former and current classmates, who considered her “weird”, and “different from others”, because of her social inhibition and psychomotor slowness, and mockingly calling her “the little painter”. She felt sexually abused by her classmates (indecent gestures of sexual nature, use of trivial language), culminating in a psycho-traumatic event after she witnessed an erotic act of someone close to her. She felt that she did not have the protection and warmth from her family, and she did not feel sufficiently understood.

After being exposed to multiple psycho-traumatic events, which can be referred to as “emotional bullying” based on the patient’s statements, in a couple of years, the patient gradually became socially withdrawn and dropped out of school in the 11th grade. At the same time, she became addicted to watching anime, stating that “I was beginning to think I was a character”. The persistence of this situation over time led to psychiatric symptoms such as depression and obsessive-compulsive symptoms that have been associated with impaired functionality and dropping out of school. The patient described herself as shy, emotionally unstable, sensitive to criticism, and perfectionist, rigid, hesitant, undecided, and being critical of herself.

At admission, clinical examination revealed very low facial expressions and gestures, distracted gaze, severe anxiety, carelessness, weekly cooperating, reduced verbal contact in simple sentences (partial verbal negativity), low tone of voice, present consciousness, and temporally-spatially oriented. In terms of perception, the patient presented with complex visual and hallucinations (“I see small, black monsters with fiery eyes telling me what to do”), auditory hallucinations (“I hear voices criticizing me”). Examination of cognitive functions indicated spontaneous and voluntary hypoprosexia, fixation hypomnesia determined by distractibility, bradyphrenia, fragmentary paranoid ideas. Volition was low/absent and activity was characterized by major difficulties in performing daily tasks. Laboratory tests, including thyroid functions, were unremarkable and within normal limits. Computerized brain tomography did not reveal any significant findings.

A few days after admission, when the patient communicated more efficiently, the psychological examination using Symptom Checklist-90 (SCL-90) showed mild levels on psychoticism scale, ambivalence, insecurity, indecision, increased vulnerability in the control and testing of reality, difficulties in differentiating the imaginary from the real, accentuated regression and dependence. The anamnesis, physical, psychiatric and psychologic examination and the lack of somatic pathology led to the diagnostic “Major recurrent depressive disorder, severe depressive episode with psychotic symptoms” according to DSM-5 criteria [1]. The patient was treated with Escitalopram 10 mg/day, Risperidone 4 mg/day, Valproic acid 600 mg/day, and Clonazepam 0.5 mg/day. A lower dose of Valproic acid was given as a mood stabilizer, and because the patient was underweight the results were positive after 2 weeks of treatment [10,11]. Similarly, 10 mg, instead of 20 mg, of Escitalopram was administered as this drug is an SSRI (selective serotonin reuptake inhibitor) with great efficacy and high doses have been associated with increased agitation and suicidal thoughts in adolescent depression [12].

During hospitalization, the psychotic symptoms decreased significantly, although the suspicion persisted, as well as the depressed mood. When communication increased and the patient became aware of the disorder, she said that before admission, she had ideas of self-depreciation and guilt and obsessive, aggressive impulses (shouting ugly words, insults, cursing people, included loved ones). At discharge, she had no psychotic symptoms, and her depression improved. The obsessive impulsive symptoms seldom appeared, and psychological examination showed a MADRS score of 20. We did not measure the obsessive-compulsive symptoms since they were manifested during the acute stage of psychotic depression at and before admission, and the patient did not report them. She reported these symptoms in the course of hospitalization when psychosis decreased significantly.

The patient was periodically evaluated in the ambulatory care service. The pharmacologic treatment was well tolerated and consisted of Escitalopram 10 mg/day, Valproic acid 500 mg/day, and Risperidone 3 mg/day for six months following discharge. Subsequently, after the patient’s symptoms improved, Valproic acid was withdrawn due to its reported teratogenic effects [13]. The patient was also referred to a psychotherapist. According to existential analysis, [14] psychotherapeutic sessions focused on the fundamental “value of life” in order to accommodate the patient with the present, by structuring her everyday life, by discussing the very structure of each day, easing the burden of tasks (the stress caused by academic papers and exams), and cultivating abilities such as drawing.

During the first six months after discharge, the patient continued to show psycho-emotional lability, paranoid ideation, social anxiety, low sociability, restricted activities (only under parental guidance) that were performed only if she did not feel pressured (i.e., having contact with people that may criticize her) or if the situation involved an effort to concentrate. She behaved childishly with an increased dependence on her mother and showed excessive insecurity regarding self-image that included gestures, words, the meaning of words, and activities.

Six months from the initial hospitalization, the patient re-enrolled in high school (12th grade). Unfortunately, she encountered many difficulties in school and performed poorly due to attention and memory disorders. Moreover, she considered that there were more interesting things to do than school (“there are other more beautiful things in life, I like to know, to discover…”). Secondly, she faced relational difficulties with colleagues and teachers, saying that “people are bad, I feel bad”. Due to these challenges, the patient failed the baccalaureate exam. However, a positive sign during this period was an increased involvement in the domestic activities with her family and regaining her ability to draw. Unfortunately, the patient could not enter a psychotherapy program like cognitive behavioral therapy [15] or dialectical behavioral therapy [16] due to psychosocial causes.

Because the patient continued to display impaired functionality in her personal and professional life, after resolving the acute psychiatric disorder, the diagnostic of a Personality disorder was considered [1]. This was based on the following: marked and persistently unstable self-image, fear of abandonment, emotional instability, chronic feeling of emptiness, and paranoid ideation. Differential diagnostic with Avoidant personality disorder (APD) [1] was considered since this personality disorder includes many criteria that correspond with the patient’s personality traits such as social inhibition, fear of being criticized, feelings of inadequacy in social relationships.

Currently, the patient manages to carry out house chores, has not been able to focus on her studies for the baccalaureate, no longer feels able to carry out more complex activities alone, does not watch anime anymore, and she draws infrequently, but with a lot of talent. The prognosis is mixed. On the one hand, it is reserved due to some degree of impaired functioning and persistence of obsessive-compulsive symptoms with no possibility for psychotherapy and, on the other hand, the prognosis could turn out to be good due to the relatively good cognitive potential and artistic talent, if allowed psychotherapy and is able to capitalize on her talent.

## 3. Discussion

This case highlights several important issues and challenges. The medical history showed that the patient had been diagnosed, at an early age, with major recurrent depressive disorder, a severe depressive episode with psychotic symptoms, and OCD accompanied by important social and school dysfunctions. One of the most important challenges of this case was establishing an accurate diagnosis and the right course of treatment. The co-occurrence of severe depressive episodes and OCD in the medical history led us to consider the common association between Bipolar Disorder (BD) and OCD as 50 to 75% of OCD cases are limited to mood episodes in BD. The majority of comorbid OCD cases appear to be secondary to mood episodes, and obsessive-compulsive symptoms occur more often, and sometimes exclusively, during depressive episodes [17] that can be as high as 75% in BD type II [18]. Notwithstanding this common association, we made the differential diagnosis between major recurrent depressive disorder with severe depressive episode psychotic elements, BP comorbidity, and OCD, based on the fact that there was no history of manic or hypomanic episode prior to the depressive episode in order to consider the diagnosis of BD. We also considered the patient’s difficulties in performing school activities, the acute and chronic stress linked with her social relations, and hypersensitivity to rejection that can lead to dissociative symptoms [19]. The patient became gradually more relaxed in the family environment, and her functioning improved significantly. It is known that dissociative states have been linked to maladaptive functioning in BPD [20] and they are likely to disrupt information processing, learning, and memory on various levels [21].

In the ambulatory care service, the differential diagnosis between BPD and Avoidant Personality Disorder (APD) was made, given that life history contained multiple negative experiences, including several with sexual content, exclusive focus on the relationships and image, mood swings, infantilism, a tendency to idealism/accusations against others, and, more importantly, immaturity and an extremely fragile self-concept because adolescence was absent from the patient’s life. Avoidance and inhibition are the most persisting and intense patient personality traits, as behavioral acts came from an incompletely developed self. Such a fragile ego that stems from multiple encountered trauma have led to splitting, a common defense mechanism characteristic of BPD [9].

When considering the diagnostic of BPD and its subtypes, the patient’s history and symptomatology did not reflect the irritable or petulant BPD subtype [22]. Despite its heterogeneity, BPD is well-characterized by “stable instability”. This means that individuals with this disorder often show instability in the areas of effect, interpersonal relationships, self-image, and behavior [23]. The current DSM-5 criteria [1] for BPD include frantic efforts to avoid abandonment, unstable interpersonal relationships alternating between idealization and devaluation, identity disturbance including unstable self-image or sense of self, impulsivity, recurrent suicidal behavior or self-harm, affective instability, chronic feelings of emptiness, inappropriate anger, and transient paranoid ideation or dissociation related to stress.

T. Millon’s 2004 classification [22] of BPD includes the “discouraged” beside “irritable”, “petulant”, and “self-destructive” types. The discouraged type represents a mixed dependent/avoidant pattern where the attachment/submissiveness is significantly represented. It is characterized by avoiding competition, having humble behavior, constantly preoccupied with insecurity, helplessness, and doubts about one’s abilities. Simple tasks seem very difficult, and life generally seems difficult and “empty”. The individual looks for evidence of affection, and if not found, becomes selfish and angry, followed by rapprochement. The obsessive-compulsive symptoms were present during the severe depressive episode and throughout this patient’s medical history. Studies showed that the comorbidity between BPD and OCD might be seen in patients with BPD [24]. Although clinical data show that the comorbidity rate of OCD in patients with BPD is less than 10% [25], more recent studies found a higher than expected overlap between OCD and BPD, reaching rates of up to 15–35% [26]. The obsessive-compulsive symptoms were isolated, thus ruling out the diagnosis of OCD in comorbidity with Major recurrent depressive disorder, as it did not meet the DSM 5 criteria [1].

Another important issue to consider in the diagnosis is the co-existence of BPD and the obsessive-compulsive symptoms due to the fact that each of them come from two different nosological group of disorders: cluster B personality disorder vs. cluster C personality disorders. There is, however, a connection between the “discouraged” type that involves features from cluster C personality disorders, that is avoidant, depressive, or dependent [27]. When considering the anamnesis, the origins of the obsessive-compulsive symptoms did not fit with the Obsessive-Compulsive Personality Disorder (OCPD) from cluster C personality disorder, but instead could derive from perfectionisms [28], a trait of personality that can, or cannot be part of OCPD. From early childhood and later on, this patient showed perfectionism, which was evident in all activities, but especially in her drawing. Perfectionism is a transdiagnostic process involved in the occurrence and maintenance of many mental health problems such as depression, bipolar disorder, and personality disorders [29]. While social perfectionism (i.e., thinking that others need self-perfection) is associated with borderline personality (Cluster B), as measured by the Millon Multiaxial Clinical Inventory [30], a study that used Minnesota Multiphasic Personality Inventory (MMPI) showed that perfectionism was mainly related to paranoid, schizotypal, antisocial, avoidant, compulsive, dependent, and passive-aggressive behavior [31] (Cluster A and C). From a psychopathological perspective, if Borderline Personality refers to a lack of an integrated self-concept and self-integration in relation to others [32], negative emotional experiences characteristic of perfectionists (shame and guilt) can be responsible for the observed relationships between perfectionism and identity processes [33,34]. This results in a synergy of similar defense mechanisms, with integration deficits or insufficient ego functions.

## 4. Conclusions

An 18-year-old female patient presents with Major recurrent depressive disorder, a severe depressive episode with psychotic symptoms. The decreased functionality before and after the depressive episode, together with the clinical symptomatology, life, and family history, led to the diagnostic of Borderline Personality Disorder-discouraged type. The diagnosis of BPD was difficult to make as it does not fall into the typical type of BPD [35], and it was derived from the association of the features of BPD, cluster B, with social perfectionism, a trait associated with cluster C of PD. Both BPD and perfectionism are associated with problems with self-conception and identity formation. This case report underlines the clinical and psychopathological particularities of BPD that, if not carefully and thoroughly evaluated, may lead to misdiagnosis. Thus far, research on BPD subtypes and their treatment have been sparse and await further validation [36].
